# Clinical and economic outcomes of adjunctive therapy with pregabalin or usual care in generalized anxiety disorder patients with partial response to selective serotonin reuptake inhibitors

**DOI:** 10.1186/s12991-014-0040-0

**Published:** 2015-01-22

**Authors:** Enrique Álvarez, José M Olivares, José L Carrasco, Vanessa López-Gómez, Javier Rejas

**Affiliations:** Department of Psychiatry, Hospital de la Santa Creu i San Pau, Universitat Autónoma de Barcelona, CiberSam, Carrer Sant Quintí, 89, 08026 Barcelona, Spain; Department of Psychiatry, Hospital Meixoeiro, Complejo Hospitalario Universitario, Vigo, Spain; Department of Psychiatry, Hospital Clínico San Carlos, Universidad Complutense de Madrid, CiberSam, Madrid, Spain; Medical Department, Pfizer, S.L.U., Alcobendas, MD Spain; Health Economics and Outcomes Research Department, Pfizer, S.L.U., Alcobendas, MD Spain

**Keywords:** Cost analysis, Generalized anxiety disorder, Pregabalin, SSRI, Partial response, Usual care, Routine medical practice

## Abstract

**Background:**

This study is done to compare the effect of adjunctive therapy with pregabalin versus usual care (UC) on health-care costs and clinical and patients consequences in generalized anxiety disorder (GAD) subjects with partial response (PR) to a previous selective serotonin reuptake inhibitor (SSRI) course in medical practice in Spain.

**Methods:**

*Post hoc* analysis of patients with PR to SSRI monotherapy enrolled in a prospective 6-month naturalistic study was done. PR was defined as a Clinical Global Impression (CGI) scale score ≥3 and insufficient response with persistence of anxiety symptoms ≥16 in the Hamilton Anxiety Rating Scale (HAM-A). Two groups were analyzed: 1) adjunctive therapy (AT) with pregabalin (150–600 mg/day) to existing therapy and 2) UC (switching to a different SSRI or adding another anxiolytic different than pregabalin). Costs included GAD-related health-care resources utilization. Consequences were a combination of psychiatrist-based measurements [HAM-A, CGI, and Montgomery-Asberg Depression Rating Scale (MADRS)] and patient-reported outcomes [Medical Outcomes Study Sleep (MOS-sleep) scale, disability (World Health Organization Disability Assessment Schedule II (WHO-DAS II) and quality-of-life (Euro Qol-5D (EQ-5D)]. Changes in both health-care costs and scale scores were compared separately at end-of-trial visit by a general linear model with covariates.

**Results:**

Four hundred eighty-six newly prescribed pregabalin and 239 UC GAD patients [mean (SD) HAM-A 26.7 (6.9) and CGI 4.1 (0.5)] were analyzed. Adding pregabalin was associated with significantly higher mean (95% CI) score reductions vs. UC in HAM-A [−14.9 (−15.6; −14.2) vs. −11.2 (−12.2; −10.2), *p* < 0.001] and MADRS [−11.6 (−12.2; −10.9) vs. −7.8 (−8.7; −6.8), *p* < 0.001]. Changes in all patient-reported outcomes favored significantly patients receiving pregabalin, including quality-of-life gain; 26.4 (24.7; 28.1) vs. 19.4 (17.1; 21.6) in the EQ-VAS, *p* < 0.001. Health-care costs were significantly reduced in both cohorts yielding similar 6-month costs; €1,565 (1,426; 1,703) pregabalin and €1,406 (1,200; 1,611) UC, *p* = 0.777. The effect of sex on costs and consequences were negligible.

**Conclusion:**

In medical practice, GAD patients with PR to SSRI experienced greater consequence improvements with adjunctive therapy with pregabalin versus UC, without increasing health-care cost. The effect of pregabalin was independent of patient gender.

## Background

Generalized anxiety disorder (GAD) is one of the most frequent mental disorders [1-3]. GAD is characterized by a chronic evolution with a relatively late-onset age (31 years as a median age) [4]. The prevalence of GAD in western societies ranges from 2.8% in Europe to 5.7% in the United States. Specifically and according to a large epidemiological report, the prevalence of GAD in Spain has been estimated to be 2% [5]. The recent existing limitations on economic resources highlight the importance of their optimal utilization and the efficient cost-consequence analysis of new treatments by health policy decision makers. The most appropriate and useful method for the estimation of economic consequences in the management of anxiety disorders is the economic analysis that take into account both the overall health-care resource costs and the acquisition cost of the prescribed drugs [6].

For anxiety disorders, their considerable chronicity of symptoms, associated comorbidities and involved physical limitations, constitute a scenario that clearly determines an important impact on work productivity as well as high medical resource use by patients. As a consequence, the economic burden of GAD in current health systems has been evaluated as highly significant [7,8]. Specifically in Spain, the gross annual direct cost of GAD has been estimated as €2 million in a total population of 3,014 patients. Half of these total direct costs were associated with prescription drugs, but the total figure includes also physical services, laboratory analysis, and fixed costs [9]. Otherwise, overall economic costs of GAD in Spain are still scarce and have not been evaluated until very recently [10]. Frequently, the most common psychoactive drugs used in the management of GAD were anxiolytics. Within the most prescribed anxiolytics, benzodiazepines have been proved both efficacious and rapid for the treatment of GAD patients [11]. Nonetheless, recent guidelines recommend the use of benzodiazepines in a short-term period in response to some limitations that have been observed such as their associated comorbidity of depressive symptoms and common adverse events like sedation; memory and psychomotor dysfunction; increase in abuse, tolerance, or dependence; and afflictive withdrawal symptomatology [12-16]. Accordingly, the Spanish Health Ministry guidelines recommended the use of benzodiazepines for a restricted period not longer than 2–4 weeks [17].

When considering a long-term approach therapy, GAD effective treatment should be focused on selective serotonin reuptake inhibitors (SSRIs), selective serotonin-norepinephrine reuptake inhibitors (SNRIs), and pregabalin as a first choice according to current European guidelines [18]. Besides the delayed onset of SSRI/SNRI therapeutic effect [18], other disadvantages include the fact that their use in monotherapy is contraindicated in GAD patients diagnosed with comorbid bipolar disorder [19-21]. On the other hand and despite potential misuse and abuse of pregabalin pointed out by some researchers [22,23], pregabalin has been proven effective and well-tolerated as a long-term therapy option in adult patients with GAD [24,25], and, as mentioned previously, it is recommended as a first choice in the therapy of GAD by European guidelines [18]. Pregabalin acts as a calcium channel modulator that allows a relatively rapid onset of action after only 1 week, as well as a similar efficacy with that of benzodiazepines and a lower rate of discontinuation than that of benzodiazepines and SNRIs [26,27].

Current health-care resource utilization and related costs may differ depending on both the exact therapies and health-care settings. It is important to consider clinical practice to attain a real-world framework which could differ extensively from clinical trial observations [28]. Focusing on daily clinical practice needs, the aim of this study was to compare the effect of adjunctive therapy (AT) with pregabalin versus usual care (UC) on health-care costs and clinical and patients consequences in GAD subjects with partial response (PR) to a previous SSRI course in medical practice in Spain.

## Methods

### Study design

This is a *post hoc* economic analysis based on data from a previous 6-month, multicenter, prospective observational study: the Amplification of Definition of Anxiety (ADAN) study carried out between October 2007 and January 2009 in outpatient mental health centers in Spain [29]. The ADAN study was designed to elucidate the effect of broadening DSM-IV criteria for GAD and was approved by the local ethics committee of the Hospital Clínico de San Carlos (Madrid). It was conducted according to the Helsinki Declaration for research in the human being. Due to the observational design of the study, only two visits (baseline and 6 months visit) were planned. The ADAN study also assessed the use of health-care resources and related costs, which were used for the present cost analysis to compare the impact of initiating treatment with pregabalin versus usual care.

### Study population

In the ADAN study, trained psychiatrists, with at least 5 years experience in mental health diseases diagnosis, were asked to select consecutive, newly diagnosed GAD patients, according to DSM-IV criteria (APA 2000) and so-called broad criteria, until the predetermined sample size was obtained [29]. Patients of both sex, aged 18 or above, who had provided their written informed consent to participate in the study, and with partial response to SSRI monotherapy were considered eligible for inclusion. Patients could also have been treated simultaneously with a benzodiazepine at standard doses. Partial response was defined as an insufficient response with persistence of anxiety symptoms >16 in the Hamilton Anxiety Rating Scale (HAM-A) [30,31] and a Clinical Global Impression scale score >3 determined at baseline visit [32]. Exclusion criteria included previous GAD diagnosis, inability or difficulty to understand patient-reported outcomes questionnaires written in Spanish, a score ≤9 point in the HAM-A scale and a score >35 in the Montgomery-Asberg Depression Rating Scale. In this analysis, only patients with a diagnosis of GAD according to DSM-IV criteria were considered eligible. Two groups (based on psychiatrist judgment) were analyzed: 1) adding pregabalin (150–600 mg/day) to existing therapy and 2) usual care (switching to a different SSRI and/or adding another anxiolytic different than pregabalin).

### Use of health-care resources and cost estimation

Health-care resource utilization associated with GAD during the previous 6-month period was retrospectively collected at baseline and at the 6-month study visit, by means of a case report form which was designed *ad hoc* for this economic analysis. Health-care resource utilization included the following: drug utilization, medical visits and hospitalizations (from patients’ medical records), and non-pharmacological treatments (recorded during patient interviews). No records of diagnostic tests were registered since this variable was considered negligible in GAD. Four categories of health-care resources utilization were established: drug treatments, non-pharmacological therapies, medical visits (psychiatrists, psychologists, general practitioner or family physicians, and emergency room visits), and days of hospitalization in psychiatry or internal medicine wards. Non-pharmacological therapies included all those treatments used in clinical practice as complementary/adjuvant (psychosocial therapy, cognitive-conductive therapy, supportive groups, and relaxation sessions) to drug treatments for GAD. Visits to primary care, emergency department, psychologist, and psychiatrist were recorded under the category “medical visits”.

Costs estimation used year 2012 prices for GAD-related health-care resources utilization under the perspective of the Spanish National Health System. The costs of drugs were estimated using retail price + taxes of the cheapest generic medication or reference price from the Spanish Pharmaceutical Drug Catalogue of 2012. The cost of non-pharmacological treatments, medical visits, and hospitalizations was obtained from the eSALUD health-care costs database for 2012 [33] updated with the 2012 health-care inflation rate [34]. Finally, some non-pharmacological resources were priced according to expert opinion and/or directly from the vendor/provider. The direct mean cost at baseline and at the 6-month visit and change from baseline was calculated by multiplying the number of resources used in each period by their respective prices.

### Clinical and patients consequences

Consequences in this study were a health profile based in the combination of psychiatrist-based measurements [Hamilton Anxiety Rating Scale, Clinical Global Impression-Improvement (CGI-I) scale, and Montgomery-Asberg Depression Rating Scale (MADRS)], and patient-reported-outcomes [Medical Outcomes Study Sleep (MOS-sleep) scale, disability (The World Health Organization Disability Assessment Schedule II (WHO-DAS II)) and quality-of-life (Euro Qol-5D (EQ-5D))] [31,35-39]. The Hamilton Anxiety Rating Scale is a 14-item scale, each with a score between 0 (absence) and 4 (severe) that explores the patients’ degree of anxiety and that includes two subscales, one for psychic symptoms and the other for somatic symptoms [40]. The CGI-I scale is a seven-point scale that evaluates the clinician’s assessment on how much the patient’s illness has improved or worsened relative to a baseline state [35]. The MADRS is a ten-item diagnostic questionnaire which psychiatrists use to measure the severity of depressive episodes in patients with mood disorders [36]. The MOS-sleep scale measures the global subjective sleep disturbance perceived by the patient based on six dimensions of sleep [37]. It consists of 12 items which form six subscales or domains: sleep disturbance, snoring, awakening with shortness of breath or headache, adequacy of sleep, daily somnolence, and sleep quantity. In addition, the MOS-sleep scale provides a summary index of sleep problems through the scores of 9 of its items: the higher the score, the worse the sleep. WHO-DAS II is a disability instrument designed based on the ICF framework, assessing six domains of functioning in daily life [38]. The WHO-DAS II is a standardized measurement of disability for use in diverse cultural settings, translated into 16 languages to date. The WHO-DAS II domains include understanding and communicating, getting around, self-care, getting along with others, life activities, and participation in society. The EQ-5D is a standardized health-related quality of life and is a generic self-reported measure of health used frequently in clinical and economic evaluations [39]. In this five-item generic measurement of health state, the degree of impairment is assessed in five domains: mobility, self-care, daily activities, pain/discomfort, and anxiety/depression. The scores of the five items may be used to calculate a utility index, or social tariff, in the range of −0.6 through 1.0, where the higher scores represent a better health state. This instrument also includes a 20-cm visual analog scale (EQ-VAS) ranging from 0 = worst imaginable health state through 100 = best imaginable health state.

### Statistical analysis

Only patients that fulfilled all inclusion criteria and none of the exclusion criteria were included in the statistical analysis. Descriptive statistics were completed for the continuous variables in the study, including the assessment of central tendency and dispersion statistics with its 95% confidence interval when possible. The Kolmogorov-Smirnov test was applied to check adjustment of data to a Gaussian distribution. For categorical variables, absolute and relative frequencies were calculated. A descriptive statistical analysis with values for mean and standard deviation (SD) was performed. Mann-Whitney *U*-test was used to compare continuous variables between the two groups of patients at baseline, while the *χ*^2^-test or the Fisher’s exact test were applied for categorical data. Differences in the use of health-care resources and costs between the two treatment groups were tested using an analysis of covariance (ANCOVA), according to the published recommendations [41] with sex, age, and baseline values as covariates. The change from baseline for quantitative variables was calculated as the final value minus baseline value and is presented as the mean value and its 95% confidence interval (CI). Adjusted changes both in health-care costs and scale scores were compared at end-of-trial visit by a general linear model with covariates. A *p* value of less than 0.05 was considered significant. Data analysis was performed using the Statistical Analysis System (SAS 9.1).

## Results

### Patient disposition and baseline characteristics

A total of 725 subjects were included in this sub-analysis sample: 486 newly prescribed pregabalin and 239 UC GAD patients [mean (SD) HAM-A 26.7 (6.9) and CGI 4.1 (0.5)]. The two study groups were well balanced with respect to demographic characteristics at baseline (Table 1). Mean age per group was 47.0 (12.6) years in the pregabalin group and 45.2 (13.6) years in the UC group. Percentage of women (72.9% and 69.1%, respectively) and work active subjects (51.8% and 49.8%, respectively) were similar in both groups. Subjects in the pregabalin group showed higher mean baseline scores of HAM-A (27.2 ± 6.6 points vs. 25.7 ± 7.2 points in the UC group; *p* = 0.015), as well as of MADRS (23.6 ± 6.9 points vs. 21.7 ± 7.5 points, respectively, *p* < 0.001). Psychiatrists’ clinical impression (CGI) was also higher in the pregabalin group: 4.23 ± 0.76 vs. 3.97 ± 0.79 (*p* = 0.000).

**Table 1:** **Demographic and baseline clinical characteristics of patients**

**Characteristic**	**Pregabalin**	**Usual care**	***p***
	***N*** **= 486**	***N*** **= 239**	
Age (years), mean (SD)	47.0 (12.6)	45.2 (13.6)	0.070
Sex (female), *n* (%)	325 (72.9%)	159 (69.1%)	0.307
Body mass index (kg/m^2^), mean (SD)	25.6 (4.0)	26.1 (4.7)	0.185
Marital status, *n* (%)			0.625
Married or with couple	307 (63.2%)	154 (65.0%)	
Single	105 (21.6%)	57 (24.1%)	
Widow/er	25 (5.1%)	9 (3.8%)	
Divorced/separated	49 (10.1%)	17 (7.2%)	
Educational level, *n* (%)			0.463
No education	15 (3.1%)	15 (6.3%)	
Primary education	183 (37.7%)	85 (35.6%)	
Secondary education	106 (21.9%)	48 (20.1%)	
Intermediate educational level	102 (21.0%)	46 (19.3%)	
Higher education (university)	75 (15.5%)	43 (18.0%)	
Others	4 (0.8%)	2 (0.8%)	
Work status, *n* (%)			0.219
Active	251 (51.8%)	119 (49.8%)	
Housewife	127 (26.2%)	58 (24.3%)	
Sick leave	47 (9.7%)	11 (4.6%)	
Unemployed	32 (6.6%)	18 (7.5%)	
Retired	19 (3.9%)	20 (8.4%)	
Does not work (students)	4 (0.8%)	7 (2.9%)	
Others	5 (1.0%)	6 (2.5%)	
Psychiatric comorbidities, *n* (%)			
Major depression	89 (18.3%)	41 (17.2%)	0.780
Panic disorder	67 (13.8%)	22 (9.2%)	0.100
Social anxiety	48 (9.9%)	16 (6.7%)	0.200
Phobias	38 (7.8%)	15 (6.3%)	0.550
Obsessive-compulsive disorder	18 (3.7%)	9 (3.8%)	0.867
Others	94 (19.3%)	41 (17.2%)	0.542
Medical comorbidities, *n* (%)			
Chronic pain	230 (47.3%)	78 (32.6%)	<0.001
Gastrointestinal disease	97 (20.0%)	53 (22.2%)	0.552
Cardiovascular disease	48 (9.9%)	24 (10.0%)	0.889
Metabolic disease	25 (5.1%)	21 (8.8%)	0.084
Genitourinary disease	26 (5.6%)	8 (3.4%)	0.312
Others	86 (17.7%)	43 (18.0%)	0.996
SSRI distribution by drug, *n* (%)			
Paroxetine	177 (36.4)	57 (23.8)	0.001
Escitalopram	98 (20.2)	49 (20.5)	0.994
Sertraline	60 (12.3)	42 (17.6)	0.160
Mirtazapine	67 (13.8)	31 (13.0)	0.852
Citalopram	52 (10.7)	21 (8.8)	0.596
Fluoxetine	39 (8.0)	39 (16.3)	0.007
Benzodiazepine use			
Users, *n* (%)	405 (83.3)	211 (88.3)	0.177
Mean (SD) # drugs	0.98 (0.58)	1.06 (0.56)	0.151
Clinical variables, mean (SD)			
HAM-A scale score	27.2 (6.6)	25.7 (7.2)	0.015
MADRS scale score	23.6 (6.9)	21.7 (7.5)	0.001
CGI-I	4.2 (0.8)	4.0 (0.8)	0.001

*SD* standard deviation; *CI* confidence interval; *HAM-A* Hamilton Anxiety Rating Scale; *MADRS* Montgomery-Asberg Depression Rating Scale; *CGI-I* Clinical Global Impression-Improvement scale.

### Anxiety and depression outcomes

**Figure 1:**
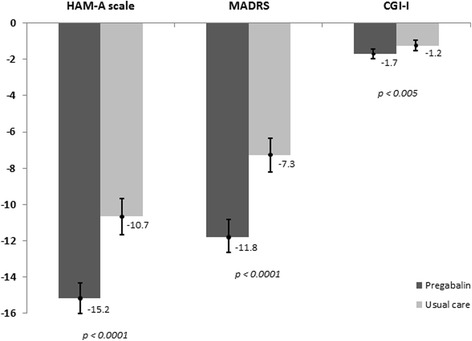
**Mean (95% confidence interval) reduction in clinical variables (HAM-A, MADRS, CGI-I) after 6 months of study in pregabalin and usual care groups.**

**Figure 2:**
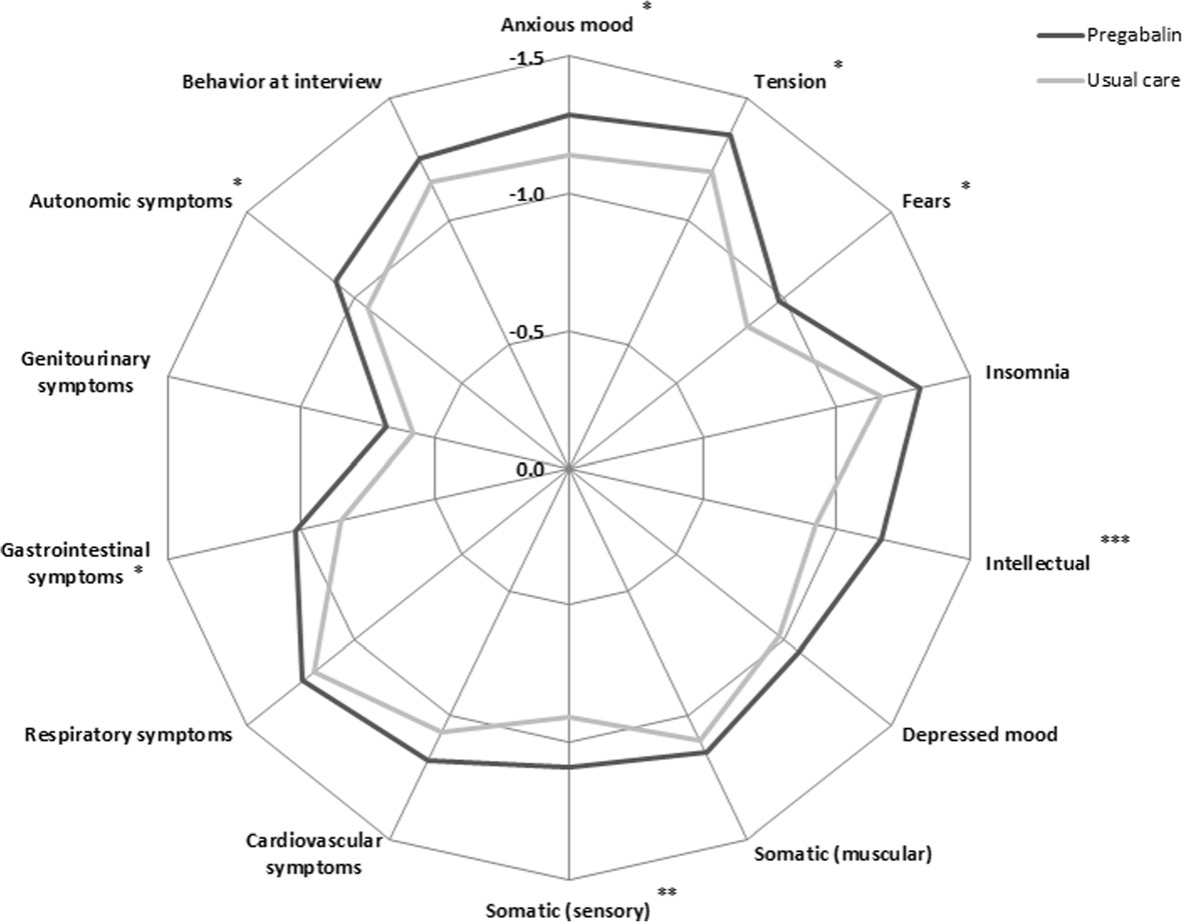
**Adjusted mean reduction in the raw score of 14 individual items of HAM-A after 6 months of study, by treatment group.** *p < 0.05; **p < 0.01; ***p < 0.001.

Adding pregabalin was associated with significantly higher benefit in anxiety and depression outcomes, as reflected by mean (95% CI) reduction vs. UC in HAM-A [−15.2 (−16.0; −14.4) vs. −10.7 (−11.8; −9.5), *p* < 0.001] and MADRS [−11.8 (−12.5; −11.1) vs. −7.3 (−8.3; −6.3), *p* < 0.001] (Figure 1). No significant differences between sexes were observed in the reduction of these variables. Similarly, the change in CGI-I scale presented significant differences between groups: −1.7 in pregabalin vs. −1.2 in UC (*p* < 0.005). Figure 2 shows in detail the specific changes in the 14 anxiety-related items included in the HAM-A. Reductions in all items favored the pregabalin group with statistically significant differences for anxious mood (*p* < 0.05), tension (*p* < 0.05), fears (*p* < 0.05), intellectual (*p* < 0.001), somatic (sensory) (*p* < 0.01), gastrointestinal (*p* < 0.05), and autonomic symptoms (*p* < 0.05).

### Patient-reported outcomes

**Figure 3:**
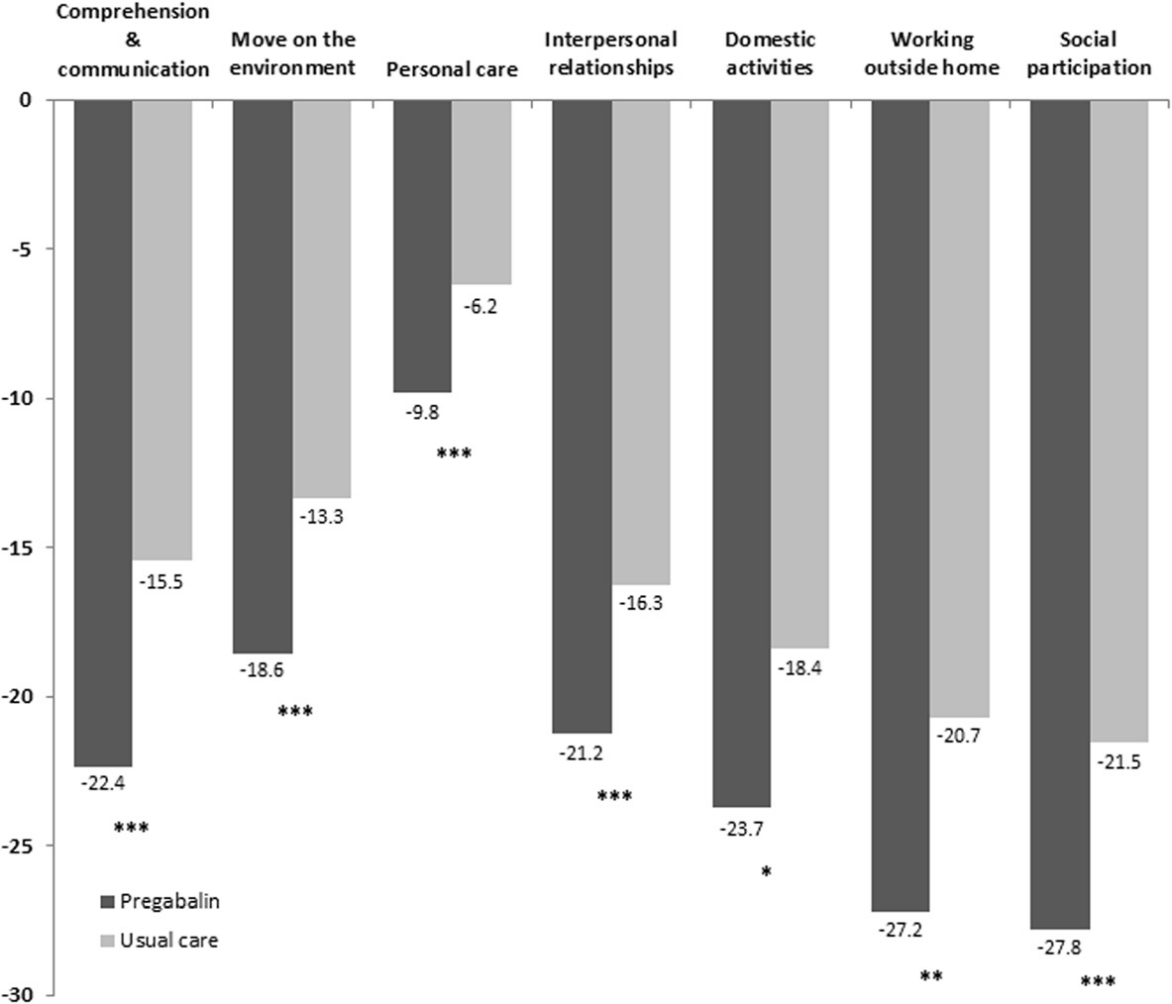
**Adjusted mean reduction in patient disability reported WHO-DAS II components after 6 months of study in pregabalin and usual care groups.** *p < 0.05; **p < 0.01; ***p < 0.001 between groups adjusted by age, sex, and baseline values.

As shown in Table 2, adjusted significant changes between visits were observed in all patient-reported outcomes. The detected improvements in sleep problems measured by the MOS-sleep scale (Table 2) and WHO-DAS II disability scale (Figure 3) favored differentially to patients in the group receiving pregabalin. Mean reduction (±95% CI) in general index sleep problems of the MOS-sleep scale was 26.4 (24.7, 28.1) in the pregabalin group and 19.6 (17.3, 22.0) in the UC group (*p* < 0.001). All items of the MOS-sleep scale reflected reductions that significantly favored the pregabalin group, except the snoring item and the optimal sleep score that accounted for non-significant lower reductions in patients treated with pregabalin (Table 2).

**Table 2:** **MOS-sleep and EQ-5D questionnaire scores by treatment group**

	**Pregabalin**			**Usual care**			***p*** **between groups**	
	***N*** **= 486**			***N*** **= 239**				
	**Baseline**	**Final**	**Change (95% CI)**	**Baseline**	**Final**	**Change (95% CI)**	**Baseline**	**Final** ^**a**^
MOS-sleep scale, mean (SD)								
Sleep disturbance (0–100)	58.1 (18.9)	27.1 (17.5)	−29.3 (−31.1, −27.4)**	50.1 (20.7)	32.0 (21.2)	−22.4 (−24.9, −19.9)**	<0.001	<0.001
Snoring while sleep (0–100)	32.5 (29.0)	23.9 (26.0)	−7.1 (−9.0, −5.2)**	30.2 (30.3)	24.7 (26.9)	−4.8 (−7.4, −2.2)**	0.175	0.142
Awakening short of breath (0–100)	39.9 (22.8)	17.7 (19.6)	−20.3 (−22.2, −18.4)**	30.6 (23.9)	20.9 (22.9)	−13.2 (−15.7, −10.6)**	<0.001	<0.001
Optimal sleep (0–1)	0.19 (0.39)	0.65 (0.48)	0.46 (0.41, 0.51)**	0.26 (0.44)	0.61 (0.49)	0.41 (0.34, 0.48)**	<0.050	0.268
Sleep quality (0–100)	28.3 (19.7)	61.3 (24.0)	31.1 (28.7, 33.5)**	35.7 (22.6)	55.1 (26.1)	22.8 (19.5, 26.1)**	<0.001	<0.001
Daytime sleepiness (0–100)	38.6 (18.6)	21.6 (15.9)	−16.0 (−17.6, −14.4)**	33.7 (19.3)	23.6 (18.5)	−12.5 (−14.6, −10.3)**	<0.001	0.006
Sleep hours per night (0–24)	5.53 (1.25)	6.91 (1.05)	1.27 (1.16, 1.38)**	5.95 (1.53)	6.91 (1.13)	1.14 (0.98, 1.31)**	<0.001	0.170
General index sleep problems (0–100)	55.0 (15.0)	27.0 (16.0)	−26.4 (−28.1, −24.7)**	47.5 (16.8)	31.2 (19.1)	−19.6 (−22.0, −17.3)**	<0.001	<0.001
EQ-5D questionnaire^b^								
Mobility	1.40 (0.50)	1.22 (0.44)	−0.17 (−0.21, −0.13)*	1.29 (0.50)	1.17 (0.39)	−0.17 (−0.22, −0.12)*	0.002	0.922
	[39.4]	[21.4]		[27.1]	[17.0]		<0.001	0.375
Personal care	1.26 (0.45)	1.09 (0.29)	−0.15 (−0.18, −0.12)*	1.19 (0.42)	1.12 (0.36)	−0.12 (−0.16, −0.08)*	0.027	0.174
	[26.0]	[8.7]		[18.4]	[10.3]		0.032	0.196
Activities of daily living	2.14 (0.50)	1.56 (0.54)	−0.52 (−0.58, −0.47)**	1.88 (0.49)	1.59 (0.58)	−0.43 (−0.51, −0.36)**	<0.001	0.047
	[93.3]	[53.3]		[81.5]	[54.7]		<0.001	0.221
Pain/discomfort	2.11 (0.63)	1.57 (0.60)	−0.51 (−0.56, −0.45)**	1.90 (0.63)	1.58 (0.62)	−0.42 (−0.49, −0.35)**	<0.001	0.043
	[85.4]	[51.5]		[74.6]	[50.8]		<0.001	0.789
Anxiety/depression	2.52 (0.52)	1.54 (0.58)	−0.94 (−1.00, −0.64)**	2.37 (0.57)	1.75 (0.61)	−0.72 (−0.80, −0.64)**	0.001	<0.001
	[98.8]	[50.0]		[95.8]	[65.7]		0.002	<0.001
Self-valuation quality of life (EQ-VAS; 0–100)	42.7 (15.5)	70.0 (17.3)	26.4 (24.7, 28.1)**	48.9 (15.7)	65.5 (18.3)	19.4 (17.1, 21.6)**	<0.001	<0.001
Health status (utility index; 0–1)	0.40 (0.30)	0.78 (0.25)	0.36 (0.34, 0.38)**	0.53 (0.31)	0.75 (0.28)	0.29 (0.26, 0.33)**	<0.001	<0.001

Values are expressed as mean (SD, standard deviation) or 95% confidence intervals (CI) unless otherwise stated.

^a^Comparison of the change from baseline between the two groups adjusted by baseline values and sex and age.

^b^The percentage of patients who still have problems (sum of categories 2 and 3) for each EQ-5D component are expressed within brackets.

*p* < 0.05, *p < 0.01, **p < 0.001 vs. baseline values.

Likewise, Figure 3 depicts the mean adjusted reduction in WHO-DAS II disability scale domains. All disability domains presented significant differences between groups in the score reduction at the final visit. The global WHO-DAS II changes for employed patients (seven domains) was −21.7 (−23.3, −20.1) in the pregabalin group and −15.3 (−17.5, −13.1) in the UC group (*p* < 0.0001), while mean reductions for unemployed patients (six domains) were calculated as −20.6 (−22.2, −19.1) in the pregabalin group and −14.7 (−16.7, −12.5) in the UC group (*p* < 0.0001).

### Quality of life

At the end of the study, changes in the majority of quality-of-life outcomes also favored significantly to patients receiving pregabalin, except for the mobility and personal care items where the differences were not significant (Table 2). Global health status estimated by the social tariff reflected a mean improvement of 0.36 (0.34, 0.38) for pregabalin patients and 0.29 (0.26, 0.33) for UC patients (*p* < 0.001) after 6 months of the study initiation. The patient’s self-assessed quality of life calculated after visual analog scale completion also shown a significant better improvement for pregabalin patients: 26.4 (24.7, 28.1) vs. 19.4 (17.1, 21.6) (*p* < 0.001).

### Health-care resource utilization

#### Pharmacological treatment

The use of pharmacological resources is detailed in Table 3. The utilization pattern of anxiolytic drugs during the study was determined by the following proportions at pregabalin vs. UC group: alprazolam (28.0% vs. 20.9%), paroxetine (24.9% vs. 25.9%), escitalopram (20.0% vs. 18.4%), lorazepam (19.1% vs. 21.8%), and diazepam (11.9% vs. 10.9%) as the most commonly used benzodiazepines/SSRI for the treatment of GAD patients with partial response to SSRI in routine medical practice (Table 3). Drugs used by less than 3% of patients are not shown. Pregabalin doses used in the study were within the recommended therapeutic range (75 to 600 mg/day) for this drug: mean (standard deviation) 186.2 (106.5) mg, for a mean duration of treatment of 5.3 (1.5) months (range: 0.1 to 6.1 months). During the study, therapeutic consequences elected by physicians in GAD patients with partial response to SSRI shown a trend towards a 1/3 reduction both in benzodiazepines and SSRI drugs independently of the considered treatment for replacement (pregabalin or other drugs).

**Table 3:** **Main pharmacological treatments during the study with pregabalin or usual care therapy**

**Pharmacological treatment**	**Pregabalin**			**Usual care**			***p*** **(percentage between groups)**
	***N*** **= 486**			***N*** **= 239**			
	***n*** **(%)**	**Dose**	**Duration**	***n*** **(%)**	**Dose**	**Duration**	
		**mg/day (SD)**	**months (SD)**		**mg/day (SD)**	**months (SD)**	
Benzodiazepines	326 (67.1)			162 (67.8)			0.867
Alprazolam	136 (28.0)	2.0 (1.3)	4.7 (2.0)	50 (20.9)	2.0 (1.4)	4.7 (1.9)	
Diazepam	58 (11.9)	10.2 (7.1)	4.4 (2.0)	26 (10.9)	12.9 (6.8)	5.1 (1.7)	
Lorazepam	93 (19.1)	2.6 (1.6)	4.7 (1.9)	52 (21.8)	1.2 (1.3)	4.8 (1.8)	
Clorazepate dipotassium	39 (8.0)	23.3 (19.7)	4.4 (2.0)	26 (10.9)	15.9 (9.5)	4.9 (1.5)	
Bromazepam	21 (4.3)	5.7 (6.4)	4.8 (1.9)	10 (4.2)	4.7 (2.1)	4.9 (1.5)	
Clonazepam	16 (3.3)	9.0 (19.2)	4.1 (2.4)	11 (4.6)	12.6 (13.0)	4.5 (2.3)	
Others	75 (15.4)	-	-	50 (20.9)	-	-	
SSRI	331 (68.1)			162 (67.8)			0.933
Paroxetine	121 (24.9)	24.5 (8.9)	5.0 (1.9)	62 (25.9)	25.6 (10.8)	5.3 (1.6)	
Escitalopram oxalate	97 (20.0)	18.2 (6.4)	5.2 (1.5)	44 (18.4)	14.9 (4.7)	4.5 (1.8)	
Mirtazapine	63 (13.0)	26.1 (9.0)	4.8 (1.8)	29 (12.1)	26.0 (6.3)	5.0 (1.3)	
Sertraline	42 (8.6)	111.9 (53.8)	5.6 (1.2)	20 (8.4)	103.9 (58.4)	5.5 (1.3)	
Citalopram	29 (6.0)	24.4 (9.2)	4.8 (1.6)	15 (6.3)	23.9 (8.4)	5.3 (1.1)	
Fluoxetine	17 (3.5)	27.7 (12.0)	4.5 (2.3)	18 (7.5)	27.0 (9.2)	4.7 (2.2)	
Others	12 (2.5)	-	-	13 (5.5)	-	-	
SNRI	106 (21.8)			39 (16.3)			0.093
Venlafaxine hydrochloride	61 (12.6)	155.4 (53.9)	5.3 (1.4)	26 (10.9)	148.0 (47.3)	5.6 (1.0)	
Duloxetine	48 (9.9)	68.7 (26.9)	5.5 (1.7)	13 (5.4)	61.8 (21.0)	4.9 (1.6)	

*SSRI* selective serotonin reuptake inhibitors, *SNRI* serotonin-norepinephrine reuptake inhibitors.

#### Non-pharmacological health-care utilization

Table 4 shows the average non-pharmacological health resources utilization at baseline and final visits of the study as well as its change from baseline. Patients under usual care have shown non-significant changes in non-pharmacological treatment use relative to study initiation, including psychosocial therapies, cognitive-behavioral therapies, supportive groups, and relaxation sessions. In the pregabalin group, only slightly higher non-pharmacological utilization was detected for psychosocial therapy and supportive group sessions (*p* < 0.05 in both). Mean medical visits, overall and by type, as well as the number of hospitalizations are shown in Table 4. After 6 months of study, the reduction in the number of all medical visits reflected significant reductions both in adjunctive therapy with pregabalin and in usual care patients. After correcting for baseline differences, the pregabalin-treated group showed non-significantly greater reductions in primary care, emergency, and psychologist visits. On the other hand, the usual care group showed slightly higher reduction in psychiatrist number of visits (−1.3 vs. −1.9; *p* = 0.113). The number of hospitalizations was similarly reduced in both groups after 6 months: −0.10 in the pregabalin-group vs. −0.09 in the UC group (*p* = 0.501).

**Table 4:** **Health resources utilization and direct costs (€) by treatment group**

	**Pregabalin**			**Usual care**			***p*** **between groups**	
	***N*** **= 486**			***N*** **= 239**				
	**Baseline**	**Final**	**Change (95% CI)**	**Baseline**	**Final**	**Change (95% CI)**	**Baseline**	**Final** ^**a**^
Resources								
Non-pharmacological treatment^b^								
Psychosocial therapy	0.1 (0.4)	0.1 (0.7)	0.07 (0.01, 0.13)*	0.1 (0.6)	0.1 (0.5)	0.01 (−0.06, 0.09)	0.122	0.226
Cognitive-behavioral therapies	0.4 (1.0)	0.3 (1.0)	0.14 (0.00, 0.30)	0.3 (1.0)	0.5 (2.0)	0.20 (0.00, 0.41)	0.441	0.642
Supportive groups	0.1 (0.4)	0.2 (0.9)	0.10 (0.01, 0.18)*	0.1 (0.7)	0.2 (0.9)	0.06 (−0.06, 0,18)	0.476	0.588
Relaxation	0.5 (1.3)	0.6 (1.7)	0.09 (−0.06, 0.25)	0.5 (1.5)	0.7 (2.3)	0.13 (−0.09, 0.34)	0.639	0.792
Number of medical visits^c^								
Primary care	11.9 (13.9)	2.4 (3.7)	−9.0 (−9.5, −8.5)***	10.4 (12.8)	2.7 (5.5)	−8.3 (−9.0, −7.6)***	0.015	0.100
Emergency department	4.6 (8.0)	0.7 (7.6)	−3.9 (−4.6, −3.2)***	3.7 (6.9)	0.3 (0.8)	−3.8 (−4.8, −2.7)***	0.078	0.833
Psychologist	3.0 (7.2)	1.8 (4.6)	−1.0 (−1.5, −0.5)***	2.8 (9.4)	2.4 (5.2)	−0.9 (−1.6, −0.2)**	0.048	0.860
Psychiatrist	5.9 (6.4)	3.9 (3.7)	−1.3 (−1.7, −1.0)***	3.9 (5.1)	3.0 (2.7)	−1.9 (−2.4, −1.3)***	<0.001	0.113
Total medical visits	25.4 (22.0)	8.7 (11.2)	−15.2 (−16.3, −14.0)***	20.7 (20.1)	7.6 (8.4)	−14.9 (−16.6, −13.2)***	0.001	0.754
Number of hospitalizations	0.2 (0.9)	0.0 (0.1)	−0.10 (−0.11, −0.09)***	0.1 (0.6)	0.0 (0.1)	−0.09 (−0.10, −0.08)***	0.134	0.501
Costs (euros year 2009):								
Total costs	2,177 (1,839)	1,565 (1,555)	−478 (−619, −338)***	1,724 (2,059)	1,406 (1,611)	−446 (−635, −257)***	<0.001	0.777
Drugs	254 (304)	534 (281)	296 (272, 319)***	204 (256)	241 (228)	27 (−5, 58)	0.001	<0.001
Non-pharmacological treatment	202 (359)	274 (577)	86 (23, 148)**	191 (379)	282 (748)	88 (3, 172)*	0.115	0.969
Medical visits	1,210 (1,253)	474 (1,002)	−660 (−753, −567)***	936 (1,136)	580 (893)	−471 (−596, −345)***	<0.001	0.013
Hospitalization	40 (254)	2 (21)	−28 (−35, −21)***	14 (149)	9 (109)	−17 (−26, −8)**	0.134	0.056

Values are expressed as mean (SD, standard deviation) or 95% confidence intervals (CI) unless otherwise stated.

^a^Comparison of the change from baseline between the two groups adjusted by baseline values and sex and age.

^b^Number of sessions per month.

^c^In a 6-month period.

*p < 0.05, **p < 0.01, ***p < 0.001 vs. baseline values.

### Direct costs

Direct costs were obtained by multiplying the unit costs by the total health-care resources used in each treatment group (Table 4). The mean direct total cost at the initiation of therapy was significantly different between the two groups of patients: €2,177 ± 1,839 for pregabalin vs. €1,724 ± 2,059 for UC group (*p* < 0.001). This was, mainly, because of a higher baseline mean cost of medical visits in the pregabalin group (€1,210 ± 1,253 vs. €936 ± 1,136, *p* < 0.001) which was the main component of the total cost representing 58% of the total cost in the pregabalin group and 54% in the UC group.

Health-care costs were significantly reduced in both cohorts yielding to similar 6-month costs; €1,565 ± 1,555 pregabalin and €1,406 ± 1,611 UC (*p* = 0.777). At the 6-month visit, medical visits were still responsible for most of the costs, but in a lower total percentage, representing now only the 30% and 41% of the total cost in the pregabalin and UC groups, respectively. The previously described reduction in the number of medical visits in both groups resulted in a significant reduction in the costs associated to this health resource (€736 in the pregabalin group and €356 in the UC group, *p* < 0.001 in both groups), although mean adjusted pharmacologic cost increased significantly in both groups of patients: €280 in the pregabalin group and €38 in the UC group (*p* < 0.001).

The overall significant increase in the drug costs in the pregabalin group of patients was compensated by significant reductions in the number of visits and almost significant in the number of hospitalizations in this group of patients. Overall, these cost components resulted in a similarly meaningful reduction of total health-care costs both in adjunctive therapy with pregabalin and UC groups: −478 vs. −446 (*p* = 0.777).

## Discussion

In this paper, we report the significant improvement in symptoms observed in GAD patients with partial response to previous SSRI treatment when changed to adjunctive therapy with pregabalin in comparison with usual care. Under current European guidelines, it is recommended that pregabalin, SSRIs, and SNRIs are used as primary therapeutic options for the treatment of GAD. In addition, it is also recommended by the Spanish Ministry of Health [17] that benzodiazepines can be used for 2–4 weeks to prevent adverse events in the medium to long term as well as afflictive withdrawal symptomatology [12-15]. Under this premise, the understanding of current therapeutic options and their optimal utilization, under an efficient cost-consequence perspective, highlights the need for an appropriate estimation of economic consequences in the management of GAD patients. This estimation must take into account overall health-care resource and drug acquisition costs, especially when partial and/or partial responses to initial therapeutic options have been demonstrated [6].

To the best of our knowledge, our study presents the first analysis of clinical and economic consequences in GAD patients with partial response to previous treatment with SSRIs in routine medical care conditions. Our data adds support to previous results on the overall economic evaluation of different therapeutic options for the treatment of GAD patients in Spain [42,43]. Furthermore, this study evaluates the effective use of pregabalin as a treatment alternative when refractory outcomes have been demonstrated, i.e., with benzodiazepines [44]. In our study, as a result of the primary endpoint, we have observed that the addition of pregabalin to usual care leads to a significant reduction in anxiety and depression symptoms in comparison to usual care (*p* < 0.0001, in both cases). These reductions have been shown to be above or equal to the 50% of the baseline values in case of pregabalin. Significantly, it has to be stated that no differences in the anxiety and depression reductions were detected between sexes, thus dismissing a differential trend in terms of this fact. These trends are in accordance with a meta-analysis of eight published trials that evaluates the efficacy and tolerability of pregabalin in the treatment of GAD [45]. In this study, pregabalin treatment resulted in an overall lowered Hamilton Anxiety Rating Scale for anxiety scores within 1 week. The observed decrease in anxiety scores of our patients also confirms the recent findings by Rickels et al. that reported a significantly greater benefit for adjunctive pregabalin compared with placebo [46]. This randomized clinical trial of GAD patients with partial response to SSRI or SNRI described a mean reduction in HAM-A total score (−7.6 for pregabalin vs. −6.4 for placebo (*p* < 0.05)) during the 8 weeks of combination treatment [46].

When considering the depression symptoms in our study, the confirmed reductions sustain the initial observations of a pooled *post hoc* analysis that reflected pregabalin reduced associated symptoms of depression despite its dose [47]. In agreement with our results, a recent open-label trial [48] found statistically significant reductions (*p* < 0.001) in depression in older patients with GAD and comorbid depression from the fourth week of treatment. Global trends in amelioration of anxiety and depression outcomes were confirmed in our study by the significant change in the patient-reported Clinical Global Impression scale that benefits adjuvant pregabalin in GAD patients with partial response to SSRI with respect to UC (*p* < 0.005).

Comparable trends were detected for pregabalin adjuvant therapy in patient-reported outcomes concerning disability state and sleep quality. Significant differences in the reduction of all disability WHO-DAS II domains were probed at the end of the study and were found to favor pregabalin therapy, both in employed and unemployed patients (*p* < 0.0001, in both cases). Improvements were substantial in cases of pregabalin treatment, with sleep amelioration and reduction in disability being of a magnitude above or equal to the 50% of the baseline values. The beneficial effects of pregabalin in disability status have been previously demonstrated in neuropathic pain patients evaluated through Sheehan Disability Inventory scale [49]. Besides, all items considered in the MOS-sleep scale, including average number of hours slept per day, presented significant higher improvements after the 6 months of the study for patients receiving pregabalin in comparison to UC. However, the only exceptions of this were the snoring score and the optimal sleep score that were lower with pregabalin but not significant. As suggested by a previous pooled analysis of seven studies of pregabalin therapy in GAD patients, 53% of the effect of pregabalin on sleep disturbance was due to a direct drug effect and 47% was due to an indirect effect mediated through prior reduction in anxiety symptom severity [50].

Improvement in sleep outcomes and reduction of daytime sleepiness in GAD patients treated with pregabalin has been also demonstrated in correlation with improvement in quality of life, according to subjective global measures, as well as with amelioration in functional impairment [50]. In our study sample of GAD patients with partial response to SSRI, pregabalin adjuvant therapy was associated with a differential 36% improved benefits in self-valuation quality of life (EQ-5D visual analogue scale) and social tariff derived from such instrument in comparison with usual care. Furthermore, improvements in sleep and disability, together with major reductions in GAD symptoms, were translated to better outcomes in some dimensions of quality of life as described by the patient. Thus, in addition to anxiety/depression, patients receiving pregabalin also showed better activities of daily living and pain/discomfort in the EQ-5D questionnaire than subjects receiving usual care. These findings are coherent with previous publication [51].

Concerning therapeutic aspects and despite the fact that all the included patients in our analysis were partial responders for SSRIs, it should be noted than more than 2/3 of the sample were still taking benzodiazepines during the last 6 months. This observation occurred independently of the pregabalin adjuvant incorporation, or lack thereof, in the usual care group. On the other hand, adding pregabalin was correlated with a near 1/3 of reduction in benzodiazepines and SSRIs prescribed by physicians. It is important to consider that under this observation, psychiatrists could fulfil the recommended therapeutic goals related with benzodiazepines early by initiating GAD therapy with pregabalin in SSRI partial responders [52].

When comparing the results of our study with a previous Spanish economic evaluation of the annual cost of GAD, confirmed by DSM-IV criteria, the mean cost of GAD in primary care was estimated at €686, from which drug costs represented 59% of the total costs [53]. This economic cost is higher than our present estimate where the pharmaceutical cost represents 34% of the overall costs in the pregabalin group and 17% in the usual care group. Overall, in our GAD patients sample with partial response to previous SSRIs, the mean total costs over 1 year decreased by €478 in the pregabalin group and by €446 in the usual care group. Although the pharmacological treatment costs per year were higher after pregabalin adjuvant incorporation (€534 vs. €241 in UC, *p* < 0.001), significant lower costs were detected for medical visits (€474 vs. €580 in UC, *p* = 0.013) and an almost significant lower cost associated with hospitalizations (€2 vs. €9 in UC, *p* = 0.056). This framework results in a similar global health-care resource costs in both groups: €1,565 in the pregabalin group and €1,406 in the UC group (*p* = 0.777).

Main weaknesses of our work should also be noted. First, although some studies show that indirect costs resulting from associated loss of productivity double the usual direct costs of GAD [54,55], our study did not consider the indirect expenses associated with neither treatment nor the out-of-pocket costs as it was performed directly from the perspective of the National Health System. Second, the non-interventional design of the original data, the ADAN study, does not allow for any inference of direct causality between GAD treatment options and full costs of the associated patient’s management. While this may be venturesome in terms of conclusive interpretations, our approach, based on real-world data, allows for health decision makers from the National Healthcare System to outline some interesting conclusions regarding the estimation of health-care resource utilization and its associated direct costs. Finally, the total study sample size included could be considered relevant in comparison with previous comparative cost analysis performed within the group of Spanish patients with refractory GAD [10,56].

## Conclusions

To conclude, our results suggested that initiating therapy with pregabalin in SSRI partial responders benefits anxiety and depression outcomes and does not imply significant increased direct costs when compared with the usual care treatment in a routine clinical practice basis. Moreover, the use of adjuvant pregabalin is associated with a lower use both of benzodiazepines and SSRIs as concomitant anxiolytic drugs. In spite of the limitations considered, the study showed that the significant higher pharmacological acquisition costs of pregabalin was compensated by improved clinical outcomes and higher reductions in non-pharmacological costs. Such a reduction in costs, such as medical visits and hospitalizations, could infer improved reductions in the overall management of GAD patients with partial response to SSRI from the National Health System perspective.

## Abbreviations

ADAN: Amplification of definition of anxiety
AT: Adjunctive therapy
APA: American Psychiatric Association
CGI-I: Clinical Global Impression-Improvement
DSM-IV: Diagnostic and Statistical Manual of Mental Disorders version IV
EQ-5D: Euro Qol-5D
GAD: Generalized anxiety disorder
HAM-A: Hamilton Anxiety Rating Scale
MADRS: Montgomery Asberg Depression Rating Scale
MOS-sleep: Medical Outcome Study Sleep
PR: Partial response
QALY: Quality-adjusted life-year
SD: Standard deviation
SSRI: Selective serotonin reuptake inhibitor
SNRI: Selective serotonin-norepinephrine reuptake inhibitor
UC: Usual care
WHO-DAS II: World Health Organization Disability Assessment Schedule II
CI: Confidence intervals
